# The effect of flipped approach in teaching critical respiratory care among nurses during the COVID-19 era

**DOI:** 10.1186/s13104-023-06311-4

**Published:** 2023-04-03

**Authors:** Leila Bazrafkan, Razieh Panahian, Nahid Zarifsanaiey

**Affiliations:** 1 Clinical Education Research Center, Shiraz University of Medical Sciences, Shiraz, Iran; 2 E-learning Department, Virtual School, Shiraz University of Medical Sciences, Shiraz, Iran

**Keywords:** Flipped approach, Learning motivation, Critical respiratory care, Knowledge, Nurses, COVID 19

## Abstract

**Objectives:**

This study investigated the efficacy of flipped approach on the nurses’ knowledge and motivation regarding critical respiratory care during the COVID-19 pandemic.

**Results:**

This pretest-posttest quasi-experimental study was performed in a hospital affiliated with Shiraz University of Medical Sciences during March-December 2021. One hundred and twenty eligible nurses selected by convenience sampling underwent a seven-day flipped approach respiratory intensive care training. The nurses’ motivation and knowledge were evaluated using the Students’ Motivation Towards Science Learning (SMTSL) and a 20-researcher-made four-option questionnaire before and two weeks after the intervention, for knowledge assessment respectively. The nurses’ knowledge and learning motivation were significantly higher after the intervention (P < 0.001). The flipped approach can improve learning motivation and nurses’ knowledge of critical respiratory care.

## Introduction

The rapid evolution of technology has led to the transformation of education and created new learning models in the past years. One technology-enabled learning approach is flipped classroom [1]. This method provides educational material outside the school using different technologies to promote self-directed and individual learning. And after that, the class will be held to group learning activities such as discussion and problem-solving in a group. The flipped classroom is an educational model in which the two elements of classroom teaching and homework are replaced [2]. Research has shown that this approach significantly affects students’ learning outcomes and motivation [3]. Learning motivation is the most crucial factor in student performance because it leads them to make the effort required to complete courses and achieve defined objectives. [4].

The COVID-19 pandemic dramatically impacted teaching practices in the last months of 2019 [5]. Nurses have a special role in providing nursing care to critically ill COVID-19 patients. In this regard, one of the major concerns of the healthcare system is providing effective educational programs for managing critically ill COVID-19 patients [6]. Unpredictable future situations are a key idea and require planning and technology. Given that Shahid Beheshti Hospital is the centre for treating COVID-19, practical training can increase service quality. In addition, no research has been done in educating nurses in the critical respiratory care in COVID-19 patients in SUMS, and an information gap is felt in this field. As a result, the present study was conducted to investigate the flipped classroom’s effect on nurses’ knowledge and learning motivation in critical respiratory care.

## Methods

### Study design

This quasi-experimental study was performed with a pretest-posttest design among nurses during March-December 2021.

### Participants

Eligibility criteria for participants:

The inclusion criteria were all nurses working at Shahid Beheshti Hospital affiliated with SUMS between one and fifteen years of experience, willingness to participate, and completing an informed consent form. All those unwilling to continue their cooperation and those who participated in a similar course were excluded.

### Teaching interventions

The intervention includes preparing teaching materials, choosing an approach, and implementing it. Two e-learning and medical education professors and a nurse with 15–20 years of experience in respiratory intensive care were responsible for generating material and training nurses about respiratory intensive care.

All the nurses undertook the flipped approach. The Learning objective of this course was to develop nurses’ knowledge and motivation about critical respiratory care. The educational content was derived from the Iranian Ministry of Health and Medical Education’s training handbook, films, and animations on respiratory intensive care.

After completing the pretest, the participants were assigned the social media (Telegram), read the course content and read six asynchronous videos. The content was uploaded every two days, and the participants were encouraged to share their experiences, discuss the topic, and ask questions. The researchers answered their questions and summarized the topics every two days. Furthermore, six two-hour in-person workshops were held over two weeks at two-day intervals. First, the instructors provided learning goals, short presentations, and open-ended questions. Then, participants engaged in small-group discussions to find answers. The instructors engaged as facilitators, guiding and encouraging individuals to participate.

The content focused on the following topics: intensive respiratory nursing care, cardiac arrhythmias, oxygen therapy, medication calculations, how to talk to hospitalized patients and write a nursing note during the COVID-19 pandemic.

## Data collection tools

The nurses’ knowledge and Learning Motivation were evaluated at baseline and two weeks after the intervention. Initially, online questionnaires were provided through Telegram to eligible nurses 24 h after completing the informed consent form. Participants who did not return the questionnaires were contacted and encouraged to do so. Notably, only one follow-up effort per participant was undertaken.

## Knowledge

The data collection tool was a 20-researcher-made four-option questionnaire for assessing nurses’ knowledge regarding critical respiratory care. Five experts in specialised nursing evaluated the qualitative content validity of the questionnaire, and Cronbach’s alpha confirmed the reliability of the questionnaire (0.83).

## Students’ motivation towards Science Learning (SMTSL)

This questionnaire was designed in 2005 by Tuan, Chin, and Shieh, with 25 items and six dimensions, including self-efficacy, active learning strategies, science learning value, performance goal, achievement goal, and learning environment stimulation. The self-efficacy scale assesses students’ beliefs about their abilities to do well in science learning assignments. The active learning strategies scale concerns students’ active participation in generating new knowledge based on their prior understanding through various strategies. Science learning value is for students to gain problem-solving skills, engage in inquiry, stimulate their thoughts, and recognize the practical application of science—students’ perceptions of important values associated with science learning. The performance goal scale addresses students’ competition with peers in the classroom and their need for teacher attention. The accomplishment goal scale measures students’ happiness with enhanced competence and achievement while learning science. The learning environment stimulation scale included factors such as program, teachers’ instruction, and student interaction that affected students’ motivation to learn science [7].

This questionnaire was developed based on the five-point Likert scale. Tuan et al. reported excellent content validity for this questionnaire (0.89) and acceptable reliability of its subscales (a = 0.70 to 0.87) [8]. Zare et al. also reported acceptable content validity for the Persian version of this questionnaire (0.83). In addition, the Cronbach’s alpha value for active strategies (0.81), science learning (0.73), performance goal (0.91), progress goal (0.76), and learning environment stimulation (0.93) domains were acceptable [9].

## Statistical methods

SPSS 22 was used to analyze the collected data, including descriptive and analytical statistical tests.The Kolmogorov-Smirnov test was used to check the distribution of data. The results of this test showed that the data distribution is non-normal, so non-parametric tests were used to check the hypotheses of this research. Wilcoxon’s non-parametric test was used to compare pre-and post-test knowledge and learning motivation scores. A P-value of 0.05 was considered the significance level.

## Sample size

One hundred and 20 nurses working at Shahid Beheshti Hospital affiliated with SUMS who enrolled in the flipped course were selected by census method during March-December 2021.

## Results

All 120 enrolled nurses completed the study and the follow-up assessment, 49 were men and 71 were women, with a mean and standard deviation of 28.43 ± 0.77 years. Table 1 shows a comparison of pre-and post-intervention nurses’ knowledge scores.

**Table 1:** Comparison of knowledge subscales of the nurses before and after the intervention

	The Rating Scale	Number	Mean rating	Z	Sig
**Intensive Respiratory Nursing Care**	Negative	22	33.50	-3.392	0.001
	Positive	47	36.64		
	Neutral	51			
**Cardiac Arrhythmias**	**Evaluation rank**	**Number**	**Mean rating**	**Z**	**Sig**
	Negative	11	23.50	-4.299	0.001
	Positive	41	27.30		
	Neutral	68			
**Medication Calculations**	**Evaluation rank**	**Number**	**Mean rating**	**Z**	**Sig**
	Negative	10	20.50	-3.998	0.001
	Positive	36	24.33		
	Neutral	74			
**Communicating Hospitalized Patients**	**Evaluation rank**	**Number**	**Mean rating**	**Z**	**Sig**
	Negative	8	22.25	-4.027	0.001
	Positive	36	22.56		
	Neutral	76			
**Write a Nursing Note**	**Evaluation rank**	**Number**	**Mean rating**	**Z**	**Sig**
	Negative	23	27.22	-1/898	0.058
	Positive	35	31.00		
	Neutral	62			
**COVID- 19**	Negative	0	0	-9.236	0.001
	Positive	106	53.50		
	Neutral	14			

Because the samples were not normally distributed, a non-parametric Wilcoxon test was used. This test compares the difference between negative, neutral and positive rating scales. Table 1 shows the effectiveness of education in the flipped classroom on nurses’ knowledge in three modes: negative, positive, and neutral. Accordingly, Z values between nurses’ all knowledge subscales were significant before and after the training. Except for COVID-19 (p = 0.001, Z = -9 / 236), this test revealed that knowledge scores were significantly higher after the intervention.

Regarding the Wilcoxon analysis results, a significant relationship was observed between the learning motivation scores before and after the intervention (Table 2).

**Table 2:** Comparison of pre and post-test learning motivation subscale-scores before and after the intervention

**Self-efficacy**	**Evaluation rank**	**Number**	**Mean rating**	**Z**	**Sig**
	Negative	24	38.54	-4.219	0.001
	Positive	63	46.08		
	Neutral	13			
**Active Learning Strategies**	**Evaluation rank**	**Number**	**Mean rating**	**Z**	**Sig**
	Negative	0	0	-7.344	0.001
	Positive	71	36.00		
	Neutral	29			
**Science Learning Value**	**Evaluation rank**	**Number**	**Mean rating**	**Z**	**Sig**
	Negative	0	0	− 0.648	0.001
	Positive	58	29.50		
	Neutral	42			
**Performance Goal**	**Evaluation rank**	**Number**	**Mean rating**	**Z**	**Sig**
	Negative	14	20.61	-2.665	0.008
	Positive	31	24.08		
	Neutral	55			
**Achievement Goal**	**Evaluation rank**	**Number**	**Mean rating**	**Z**	**Sig**
	Negative	0	0	-7.248	0.001
	Positive	69	35.00		
	Neutral	31			
**Learning Environment Stimulation**	**Evaluation rank**	**Number**	**Mean rating**	**Z**	**Sig**
	Negative	0	0	-7.145	0.001
	Positive	67	34.00		
	Neutral	33			
**Total**	**Evaluation rank**	**Number**	**Mean rating**	**Z**	**Sig**
	Negative	1	5	-8.579	0.001
	Positive	97	49.96		
	Neutral	2			

Table 2, shows the effectiveness of education in the flipped classroom on nurses’ learning motivation in three modes: negative, ineffective, and positive. Accordingly, Z values between nurses’ all learning motivation subscales were significant before and after the training.

## Discussion

This study evaluates the influence of flipped classrooms on nurses’ knowledge and motivation to learn concerning critical respiratory care. According to the results, this strategy enhanced the learning and motivation of the nurses.

The findings of the present study are consistent with previous findings addressing the effects of flipped classrooms on academic performance and development of higher-level thinking abilities [10], self-directed learning and metacognitive awareness [11], learning effectively, and motivation for learning on nurse [12]. On the other hand, some studies show the same effectiveness for flipped and traditional methods in knowledge, self-efficacy and motivation [6]. According to several experts, suitable teaching approaches are strongly tied to efficacy. As a result, online, face-to-face, or flipped delivery techniques alone do not affect audience learning [13]. The current study first offered the content for nurses via social media to facilitate independent and flexible learning. They were then invited to discuss the topic to reinforce collaborative learning.

Furthermore, they participated in workshops to strengthen group conversations regarding the issues. Therefore, the participants had a high level of cooperation and engagement. This strategy incorporates both self-directed and group activities. Consequently, these benefits may increase the efficacy of training using a flipped method.

Moreover, consistent with similar findings addressing the benefits of the flipped classroom on active learning strategies, academic progress, group interaction, the outcome of cognitive learning subscales, and the overall motivation of learners during the learning process is the result of the current study [14–17]. According to Self-Determination Theory (SDT), Autonomy and Active Involvement can increase motivation in flipped classrooms by allowing students to provide their learning approach. Thus, the flipped pedagogy encourages students to acquire new knowledge [18].

The present study’s teaching approach was based on active and interactive learning techniques [19]. The flipped classroom may also give students the impression that the instructor is more available individually and in group discussions since students’ participation is enhanced. Our research demonstrated that students could think critically, solve problems, present their findings, and discuss concepts [20–22].

Given that nurses play a crucial role in the critical respiratory care of COVID-19 patients, practical training in this field can increase their effectiveness. The findings of this study indicated that a flipped educational program could improve nurses’ knowledge and learning motivation.

## Limitation

The study also had some limitations; the sample was obtained from a local hospital, and the evaluation was conducted two weeks after the training was completed. In light of these constraints, future studies should investigate the flipped learning strategy over a longer time frame and with a bigger sample size.

## Data Availability

The data supporting this study’s findings are available from the corresponding author on request.
